# Metagenomic of clinically diseased and healthy broiler affected with respiratory disease complex

**DOI:** 10.1016/j.dib.2018.05.010

**Published:** 2018-05-08

**Authors:** J.G. Patel, B.J. Patel, S.S. Patel, S.H. Raval, R.S. Parmar, D.V. Joshi, H.C. Chauhan, B.S. Chandel, B.K. Patel

**Affiliations:** a Department of Veterinary Pathology, College of Veterinary Science and Anima Husbandry, Sardarkrushinagar Dantiwada Agricultural University, Sardarkrushinagar, Gujarat, India; b Department of Veterinary Microbiology, College of Veterinary Science and Animal, Husbandry, Sardarkrushinagar Dantiwada Agricultural University, Sardarkrushinagar, Gujarat, India; c Department of Animal Biotechnology, College of Veterinary Science and Animal, Husbandry, Sardarkrushinagar Dantiwada Agricultural University, Sardarkrushinagar, Gujarat, India

## Abstract

In recent past, the respiratory infection has emerged as a great challenge to the poultry farmers. Various pathogens including Avian pneumovirus (APV), Avian influenza virus (AIV), Infectious bronchitis virus (IBV) and Newcastle disease virus (NDV), *Avibacterium paragallinarum, Ornithobacterium rhinotracheale* (ORT), *Mycoplasma synoviae* (MS), *Mycoplasma gallisepticum* (MG) and *Avian pathogenic Escherichia coli* (APEC) are involved in the respiratory disease complex in birds [1], [2] (Bradbury, 1984; Roussan et al., 2008). Hence, respiratory disease complex is the most serious disease affecting to poultry and causes heavy economic losses in the poultry industry worldwide [3] (Murthy et al., 2008). In recent years, metagenomics is powerful analyzing tool for detection of pathogens directly from clinical samples without any prior knowledge of the organism in a given sample [4], [5] (Schuster, 2008; Pereira et al., 2010). High throughput Next-Generation-Sequencing technology was used for sequencing the isolated genomic DNA. These data provides an insight about taxonomic and functional status of microorganisms responsible for causing respiratory infection in broiler. The data of these metagenome are available in the BioSample Submission Portal as Bioproject PRJNA339659 and SRA accession number SRR5997823, SRR5992854, SRR6037376, SRR6024702, SRR6012248 and SRR6008913.

**Specifications Table**

**Table:** 

Subject area	Veterinary
More speciﬁc subject area	Metagenomics, Pathology
Type of data	Metagenomics raw data
How data was acquired	Ion Torrent PGM Next Generation Sequencing Platforms
Data format	Fastq ﬁle
Experimental factors	Six respiratory lavage collected from healthy and diseased broiler.
Experimental features	Freshly collected respiratory lavage was used for total DNA extraction and whole genome shotgun (WGS) sequencing.
Data source location	In and around Palanpur, Gujarat, India
Data accessibility	These metagenome data are available in the NCBI BioSample Submission Portal as Bioproject PRJNA339659 and SRA accession number SRR5997823, SRR5992854, SRR6037376, SRR6024702, SRR6012248 and SRR6008913. (https://www.ncbi.nlm.nih.gov/sra/?term=PRJNA339659). Study ID in the MG-RAST metagenomic analysis server are e2112c56076d676d343732323631372e33, ebfbacd9ba6d676d343734383230392e33, 5a3850c1b36d676d343734383231342e33, d4d760d3f76d676d343732323339332e33, befd47c9cc6d676d343734383539362e33, 7c395102e76d676d343734383539352e33

**Value of the data**

• These data represents the first WGS metagenomic sequences of genomic DNA isolated from respiratory tract of broiler.
• Metagenome data are used to identify different pathogens responsible for causing respiratory disease complex.
• These metagenome are valuable for the study and comparison of microbial communities among different types of birds.
• The additional metagenome data will provide information about virulence genes of respiratory pathogens for prevention and control of disease.

## 1 Data

Metagenomics is newer technique to overcome the limitations of culture dependent microorganism studies [5]. Metagenomics can be performed directly from clinical samples [6] or even single cells and can detect pathogens at very low abundance [7] . It is useful in identifying novel species and strains [8], outbreaks [9] and complex diseases. The present work was carried out to detect pathogens responsible for respiratory disease complex in broiler. The studies described microbial diversity, abundance, taxonomic classification and functional analysis of pathogens in the broiler affected with respiratory infection. There are several pathogens were detected by metagenomics, of which *Mycoplasma synoviae*, *Mycoplasma gallisepticum, Avibacterium paragallinarum, Ornithobacterium rhinotracheale Escherichia coli* were most important pathogens for respiratory disease complex.

## 2 Experimental design, materials, and methods

### 2.1 Sample collection

Approximately 500 µl to 1.2 ml of respiratory lavage was collected from each bird and transferred to a sterile 1.5-ml microcentrifuge tube (Eppendrof, Germany) and stored at -20°C until further processed for genomic DNA extraction.

### 2.2 DNA extraction and metagenome sequencing

Genomic DNA (gDNA) was extracted by using a commercially available DNA isolation kit (DNeasy blood and tissue kit, Qiagen) according to the manufacturer's instructions. Genomic DNA concentration was measured using Qubit® dsDNA HS Assay Kit (Life Technologies). Total 100 ng of DNA was used to prepare the library using the Ion Xpress™ Plus Fragment Library Kit (Cat. No. 4471269) and Ion Xpress™ Barcode Adapters 1–16 (Cat. No. 4471250). The final library was used for emulsion PCR, enrichment and sequencing. The Ion PGM™ Hi-Q™ Sequencing Kit and 318 chip were used for sequencing reactions, following the recommended protocol.

### 2.3 Data accessibility

The metagenome sequences of broiler have been deposited in the National Center for Biotechnology Information (NCBI) BioSample Submission Portal as Bioproject PRJNA339659 and SRA accession number SRR5997823, SRR5992854, SRR6037376, SRR6024702, SRR6012248 and SRR6008913. (https://www.ncbi.nlm.nih.gov/sra/?term=PRJNA339659).
