# Supplementary material for: Metagenomic of clinically diseased and healthy broiler affected with respiratory disease complex
Source: Data Brief. 2018 May 8;19:82–5. doi: 10.1016/j.dib.2018.05.010 (PMC5993000; doi:10.1016/j.dib.2018.05.010)
Supplement: Supplementary file 1 — Supplementary material [file mmc1.docx]

**Conflicts of interest:**

The authors confirm that there are no known conflicts of interest associated with this publication and there has been no significant financial support for this work that could have influenced its outcome.
